# Extraordinary Response to Mitotane Monotherapy in Advanced Aggressive Adrenocortical Carcinoma

**DOI:** 10.1210/jcemcr/luaf174

**Published:** 2025-07-31

**Authors:** Maria Elena Aloini, Pina Lardo, Roberta Maggio, Iolanda Matarazzo, Alfredo Berruti, Antonio Stigliano

**Affiliations:** Department of Endocrinology, Department of Clinical and Molecular Medicine, Sant'Andrea University Hospital, Faculty of Medicine and Psychology, “Sapienza” University of Rome, Rome 00189, Italy; Department of Endocrinology, Department of Clinical and Molecular Medicine, Sant'Andrea University Hospital, Faculty of Medicine and Psychology, “Sapienza” University of Rome, Rome 00189, Italy; Department of Endocrinology, Department of Clinical and Molecular Medicine, Sant'Andrea University Hospital, Faculty of Medicine and Psychology, “Sapienza” University of Rome, Rome 00189, Italy; Department of Surgical and Medical Sciences and Translational Medicine, Sant’Andrea University Hospital, “Sapienza” University of Rome, Rome 00189, Italy; Department of Oncology, Spedali Riuniti University of Brescia, Brescia 25123, Italy; Department of Endocrinology, Department of Clinical and Molecular Medicine, Sant'Andrea University Hospital, Faculty of Medicine and Psychology, “Sapienza” University of Rome, Rome 00189, Italy

**Keywords:** adrenocortical carcinoma, mitotane, etoposide-doxorubicin-cisplatin (EDP), surgery

## Abstract

Adrenocortical carcinoma (ACC) is an aggressive tumor; the 5-year overall survival rate for advanced disease is <15%. First-line therapy for advanced disease is mitotane, either as monotherapy or in combination with chemotherapy. Overall survival after these strategies is comparable when used in the appropriate subgroup of patients. Mitotane monotherapy is usually reserved for biologically low-risk tumors, with a reported partial response rate of 13% to 31%. Typically, responses are observed when mitotane plasma levels are >14 mg/L, but sometimes partial and complete responses have been reached with lower levels. No single clinical or pathological factor has been extensively validated to predict the response to mitotane monotherapy. We describe the case of a 45-year-old patient with metastatic, rapidly growing, unresectable ACC who had a remarkable response to mitotane monotherapy. After 4 months, a 45% reduction of the primary tumor and regression of lung metastases were observed, despite plasmatic mitotane levels of 5 mg/L. Mitotane may represent an effective therapy in selected cases of advanced ACC and despite plasma levels <14 mg/L.

## Introduction

Adrenocortical carcinoma (ACC) is a rare endocrine malignancy with a generally poor prognosis. Over 50% of cases are diagnosed when advanced (unresectable or metastatic) [1], with a 5-year overall survival (OS) rate of 40% to 50% and <15% for European Network Study of Adrenal Tumors (ENSAT) stage III and IV, respectively [2, 3]. For patients with advanced ACC, mitotane is an important treatment option, either as monotherapy (in those with low disease burden or less aggressive disease) or combined with chemotherapy. The goals of treatment are controlling tumor growth and hormone secretion [1]. When used in and appropriate group of patients, OS following etoposide, doxorubicin, cisplatin + mitotane (EDP + M), and mitotane monotherapy are 14 to 16 months and 18 months, respectively [4, 5]. However, there are documented cases of disease stabilization and even complete remission many years postdiagnosis [6], and some patients become long-term survivors. Better survival is observed in patients with resectable, oligometastatic tumors and long recurrence-free intervals [1, 5-7].

Mitotane monotherapy is typically reserved for patients with low-risk disease and more favorable prognostic factors (low tumor grade, R0 resection status, younger age, low tumor burden, and absence of hormone-related symptoms) [1, 2]. The partial response rate to mitotane monotherapy varies from 13% to 31% [8, 9], with complete response being rarer [10]. Responses often occur in patients who achieve mitotane blood levels >14 mg/L [8, 11, 12], although several studies reported antitumor activity even with serum mitotane values below the therapeutic range [13]. Mitotane efficacy is usually limited in time, and the majority of patients progress and require second-line therapy [such as streptozocin (± mitotane) or gemcitabine + capecitabine (± mitotane)], if clinical trials are not available [1, 7, 14]. Despite some clinical trials highlighting the appropriate management of ACC in different settings [15, 16], currently no single clinical or pathological factor has been widely validated to accurately predict response to mitotane.

Here, we present the case of a patient with metastatic, rapidly growing, unresectable ACC who responded remarkably well to mitotane monotherapy.

## Case Presentation

**Figure 1.**
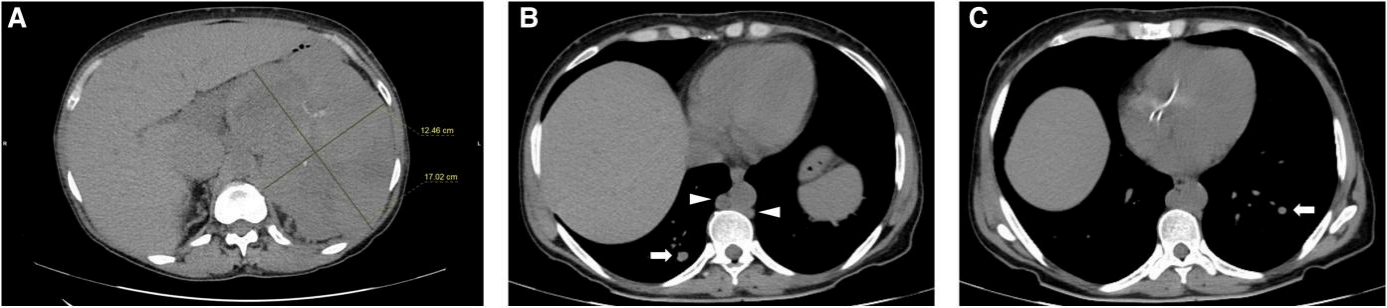
A non-contrast-enhanced abdominal computed tomography scan performed at the diagnosis shows a large solid mass of 17 cm with homogeneous and inhomogeneous areas located in the left adrenal lodge, encompassing the ipsilateral kidney (A), multiple bilateral lung nodules (white arrows), and para-aortic lymphadenopathy (white arrowheads) (B, C).

A 45-year-old woman with persistent lower limb edema, had an abdominal computed tomography (CT) scan revealing a left adrenal mass of 17 cm, encompassing the ipsilateral kidney (Fig. 1A). Forty days later, the lesion measured 20 cm and extended into the contralateral adrenal lodge, displacing adjacent organs and splanchnic vessels. The CT also showed para-aortic lymphadenopathy, pulmonary embolism, and multiple bilateral pulmonary nodules (Fig. 1B,C); ^18^fluorodeoxyglucose-positron emission tomography revealed increased uptake in the left adrenal gland, lymph nodes, and lungs. An adrenal biopsy confirmed the ACC diagnosis (no information on Ki67 or mitotic count was provided).

The patient's Eastern Cooperative Oncology Group (ECOG) performance was 0, without clinical signs of hypercortisolism or hyperandrogenism. She had no significant past medical history and was on no chronic medications, except for heparin prescribed for the pulmonary embolism. She reported a history of smoking.

## Diagnostic Assessment

Hormonal assays revealed hypersecretion of androgens and cortisol (Table 1). The case was discussed within the multidisciplinary team and systemic chemotherapy and EDP + M was proposed.

**Table 1.** Pre- and post-mitotane treatment hormonal tests, with reference ranges

	Before treatment	After 6 months mitotane	Reference range
DHEAS (serum)	**>1500 µg/dL** **(SI: > 40.5 µmol/L)**	32.9 µg/dL (SI: 0.89 µmol/L)	30-182 µg/dL (SI: 0.81-4.91 µmol/L)
17OHPG (serum)	**1.8 ng/mL** **(SI: 54.5 nmol/L)**	0.3 ng/mL (SI: 0.9 nmol/L)	0.08-1.3 ng/mL (SI: 0.24-3.94 nmol/L)
Testosterone (serum)	**2.8 ng/mL** **(SI: 9708.2 pmol/L)**	0.1 ng/mL (SI: 346.72 pmol/L)	0.1-0.57 ng/mL (SI: 346.72-1976.3 pmol/L)
Androstenedione (serum)	0.4 ng/mL (SI: 1.4 nmol/L)	0.1 ng/mL (SI: 0.35 nmol/L)	0.3-10 ng/mL (SI: 1.05-35 nmol/L)
Cortisol Am (serum)	15.79 μg/dL (SI: 436 nmol/L)	3.71 μg/dL (SI: 102.5 nmol/L)	3.66-19.4 μg/dL (SI: 101-536 nmol/L)
ACTH (serum)	9.6 pg/mL (SI: 2.11 pmol/L)	**169.6 pg/mL** **(SI: 37.3 pmol/L)**	4.7-48.8 pg/mL (SI: 1.03-10.7 pmol/L)
UFC (urine)	**333 µg/24 hours** **(SI: 918.75 nmol/day)**	53 µg/24 hours (SI: 146.23 nmol/day)	4.30-176 µg/24 hours (SI: 11.9-485.6 nmol/day)
Aldosterone (serum)	86 pg/mL (SI: 0.24 nmol/L)	24.3 pg/mL (SI: 0.07 nmol/L)	17.6-232 pg/mL (SI: 0.05-0.64 nmol/L)
PRA (plasma)	1.8 ng/mL/h (SI: 1.8 ng/mL/h)	3.2 ng/mL/h (SI: 3.2 ng/mL/h)	0.06-4.69 ng/mL/h (SI: 0.06-4.69 ng/mL/h)
Metanephrines (24-hour urinary fractionated)	0.15 mg/24 hours (SI: 760.5 nmol/day)		0.05-0.34 mg/24 hours (SI: 25.4-172.4 nmol/day)
Normetanephrine (24-hour urinary fractionated)	0.07 mg/24 hours (SI: 38.2 nmol/day)		0.08-0.45 mg/24 hours (SI: 43.7-245.6 nmol/day)
DST 1 mg (serum cortisol Am)	**14.2** **μg/dL** **(SI: 393 nmol/L)**		<1.8 μg/dL (SI: <50 nmol/L)

Abnormal values are in bold. Pretreatment, hyperandrogenism was documented by serum DHEAS, 17OHPG, and testosterone levels and autonomous cortisol secretion by the DST 1 mg; post-mitotane treatment (6 months), a reduction in the size of the pulmonary nodules and the left adrenal mass was accompanied by a remarkable reduction in levels of serum glucocorticoids, UFC, and serum androgens. Fractionated urinary metanephrines and DST 1 mg were not assessed posttreatment.

Abbreviations: 17OHPG, serum 17 hydroxyprogesterone; DHEAS, serum dehydroepiandrosterone sulphate; DST, 1 mg. 1 mg dexamethasone suppression test; PRA, plasma renin activity; UFC, urinary free cortisol.

## Treatment

Mitotane was started at a dose of 1.5 g/day, alongside EDP. Cortisone acetate was also prescribed at 62.5 mg/day. After the first chemotherapy administration, she developed severe, persistent acute kidney injury [creatinine 3.9 mg/dL (SI: 344.76 µmol/L)] [reference range, 0.5-1.1 mg/dL (SI: 44-97 µmol/L)]; glomerular filtration rate 13.1 mL/min (SI: 0.22 mL/s) [reference range, 90-131 mL/min (SI: 1.5-2.19 mL/s)], leading to the decision to continue with mitotane alone, progressively increasing the dose. Additionally, metyrapone was prescribed, then gradually discontinued, when cortisol levels normalized.

## Outcome and Follow-up

**Figure 2.**
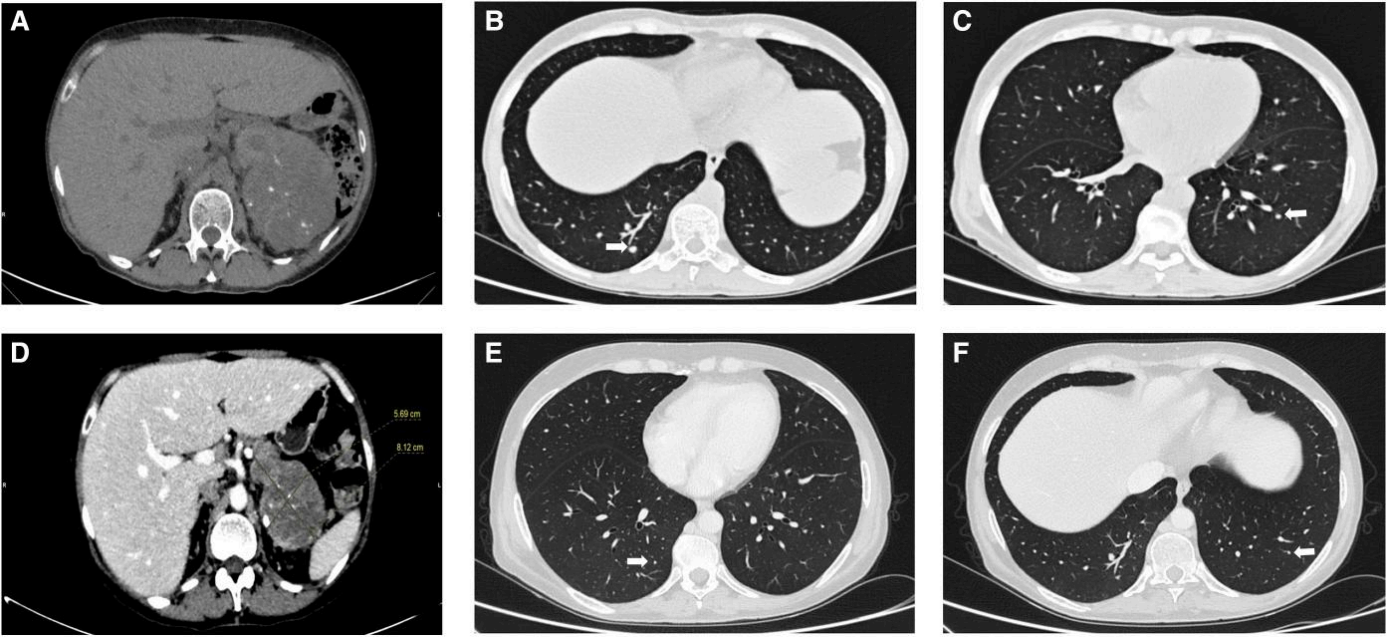
A contrast-enhanced computed tomography scan documented a reduction of the primary tumor (maximum diameter of 11 cm) (A) and in size of lung bilateral metastatic foci 4 months after starting mitotane (B, C). Follow-up imaging performed after 8 months after mitotane treatment showed an impressive regression of adrenocortical carcinoma (maximum diameter 8 cm) (D) and the lung metastatic nodules (white arrows) (E, F).

**Figure 3.**
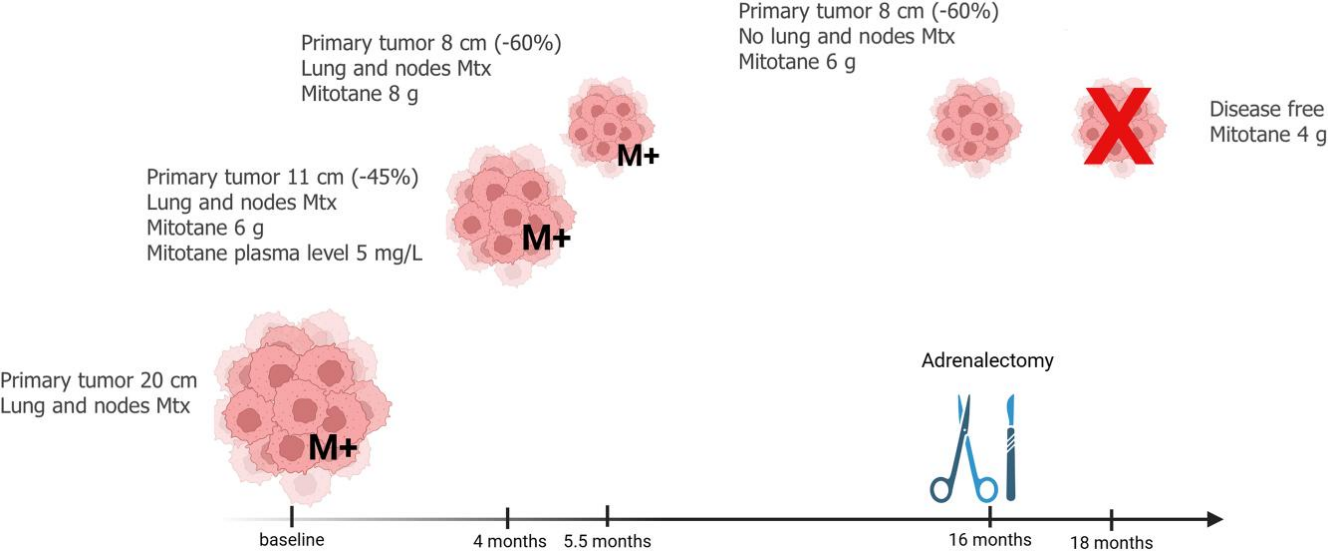
Response to mitotane treatment [tumor dimensions and presence of metastases (M+)] over time (expressed as months) from the start of treatment to date.

After 4 months, a CT scan documented a 45% reduction of the left adrenal mass (11 cm) and in the size of the pulmonary nodules; mitotane blood levels were 5 mg/L (drug dose 6 g/day). The mitotane dose was progressively increased (maximum dose 8 g/day), until therapeutic levels were achieved after an additional 45 days. After another 4 months, further reduction of the pulmonary nodules and the adrenal mass (8 cm; 60%) was observed (Fig. 2), with mitotane levels of 35.3 mg/L (consequently, to avoid side effects, the mitotane dose was reduced appropriately). Sixteen months since the start of mitotane, a CT no longer showed lymphadenopathy and only a 3 mm pulmonary nodule persisted. Fluorodeoxyglucose uptake of the left adrenal lodge had also significantly reduced (Standardized Uptake Value 5 vs 12), and the lesion did not involve the contralateral kidney anymore. (Fig. 3). The adrenal mass was then removed, and a pathology report confirmed the ACC diagnosis (Weiss score 7, Ki67 6%, R1).

During follow-up, the patient experienced the following adverse events (according to the Common Terminology Criteria for Adverse Events 5.0): grade 2 anorexia and weight loss, grade 2 anemia, grade 2 gamma-glutamyl-transferase increase, grade 1 transaminase increase, grade 3 hypercholesterolemia, grade 1 hypertriglyceridemia, grade 2 depression, and central hypothyroidism. The patient's ECOG performance status remained 0.

She now (18 months since EDP administration) remains on mitotane monotherapy at 4 g/day; her mitotane level is 8.9 mg/L and creatinine 1.2 mg/dL (SI: 106.1 µmol/L); her glomerular filtration rate 54.5 mL/min (SI: 0.91 mL/s), and she has resumed her normal work activities.

## Discussion

The therapeutic response achieved after mitotane was remarkable, especially given some unfavorable prognostic characteristics that led to the initial choice of first-line chemotherapy (unresectable tumor, rapid growth, hypercortisolism, tumor-related symptoms at diagnosis) [2]. Moreover, the adrenal biopsy is an additional widely recognized negative prognostic factor, potentially associated with metastases spreading [4]. Mitotane, a highly lipophilic drug, has low oral bioavailability, a large volume of distribution, and a long half-life. It generally takes 3 to 5 months to achieve therapeutic plasma levels [17], making its use in monotherapy typically inadequate for controlling aggressive, highly proliferative tumors [10]. Since the primary tumor was unresectable, we opted for EDP + M, but chemotherapy was discontinued because of acute kidney injury. Surprisingly, after 4 months of mitotane monotherapy, a partial response was observed, despite mitotane levels being below the therapeutic range.

Studies evaluating mitotane monotherapy in patients with advanced ACC report partial response rates of 13% to 31% [8, 9]. Complete responses are much rarer [10]. Most of the responses are observed when mitotane levels are >14 mg/L [8, 11, 12], but patients with metastatic ACC achieving great responses with lower plasma levels have been reported [9, 12]. In fact, the therapeutic threshold of 14 mg/L is supported by low-level evidence [13]. Despite the use of mitotane being know for a long time, the adrenolytic mechanism underlying mitotane action is still largely unknown and seems to require activation at the adrenal level [18]. No preclinical evidence suggests that mitotane lacks antitumor activity at concentrations <14 mg/L [19]. Higher plasma concentrations have been related to greater OS [9, 19, 20]; however, it is the duration of exposure to a therapeutic level (time in the target range), rather than the maximum absolute plasma concentration achieved, that determines mitotane efficacy and better predicts OS [21]. Probably, mitotane plasmatic levels do not fully reflect its intratumoral concentration, and patients who respond better achieve higher concentrations at the tumor level due to a greater intra-adrenal activation.

Some prognostic parameters have been identified in patients with ACC (R0 resection status, lower ENSAT stage, low grade/lower Ki67, low tumor burden, younger age, absence of symptoms at diagnosis, absence of cortisol secretion) [2, 5]. A better response to mitotane monotherapy has been reported for patients with low disease burden (<10 lesions) and longer recurrence-free survival (>360 days) after initial surgery, better performance status at diagnosis, and nonsecreting tumors [7, 9]. However, not all patients achieving a complete response fit these parameters. In particular, the absence of hormonal secretion at diagnosis emerges as a less significant factor in predicting the clinical response; many patients achieving a complete response had cortisol and/or androgen-secreting tumors [19, 22]. Recently, the S-GRAS score was proposed as a simple tool to preselect operated patients who might benefit from different adjuvant strategies. In particular, the authors highlighted how a score of 4 to 5 predicts longer recurrence-free survival after mitotane monotherapy, while adjuvant chemotherapy would be needed for patients with higher scores. Note that not all components of the score carry the same weight: ENSAT stage and Ki67 would have greater strength in prognosis prediction [23].

The rapid initial growth of the tumor remains an open question in this case. Surprisingly, the Ki67 value on the pathology report was 6%, leading us to hypothesize that this low value might be a consequence of the action of mitotane on the tumor. To confirm our hypothesis, we asked for a revision of the adrenal biopsy (unfortunately on a poor original sample), but a low Ki67 (4%) was confirmed. However, the low Ki67 would be consistent with the great response obtained after mitotane monotherapy and with a better prognosis [23], even if it hardly explains the initial rapid growth of the tumor.

New biomarkers are needed to more precisely predict responses to different therapeutic strategies. Recently, the role of the neutrophil-to-lymphocyte ratio in predicting a better response to mitotane monotherapy in advanced ACC has been suggested, with a neutrophil-to-lymphocyte ratio ≥ 5 correlating with a poorer response and lower OS [20]. Moreover, specific combinations of certain single nucleotide polymorphisms in the cytochrome P450 2W1 and 2B6 might predict the response to mitotane monotherapy [24]; genotyping of these single nucleotide polymorphisms could be useful for selecting responders to mitotane monotherapy. Unfortunately, these data were not available for our patient and still need wider clinical validation.

In conclusion, this case highlights that mitotane monotherapy could be effective in selected cases of rapidly growing advanced ACC, regardless of plasma levels achieved. Considering that there are no widely validated predictors of mitotane efficacy, we recognize the limitation of a single case report. Future studies in this direction could help to identify patients who will benefit from different treatment strategies in the setting of advanced ACC.

## Learning Points

- Mitotane monotherapy may be effective in selected cases of rapidly growing advanced ACC.
- Mitotane therapeutic efficacy does not always correlate with the achievement of plasma levels >14 mg/L; it might be influenced by intratumoral concentration rather than plasma levels alone.
- For treatment with mitotane, a reasonable goal is to achieve a well-tolerated and stable in the long term drug level.

## Data Availability

Original data generated and analyzed during this study are included in this published article.
